# Accuracy and efficiency of dynamic navigated root-end resection in endodontic surgery: a pilot in vitro study

**DOI:** 10.1186/s12903-024-04306-6

**Published:** 2024-05-19

**Authors:** Si-Min Liu, Li Peng, Yi-Jiao Zhao, Bing Han, Xiao-Yan Wang, Zu-Hua Wang

**Affiliations:** 1 Department of Cariology and Endodontology, Peking University School and Hospital of Stomatology & National Center for Stomatology & National Clinical Research Center for Oral Diseases & National Engineering Research Center of Oral Biomaterials and Digital Medical Devices, Beijing, PR China; 2 Fourth Clinical Division, Peking University School and Hospital of Stomatology & National Center for Stomatology & National Clinical Research Center for Oral Diseases & National Engineering Research Center of Oral Biomaterials and Digital Medical Devices, Beijing, PR China; 3 Department of General Dentistry II, National Center for Stomatology & National Clinical Research Center for Oral Diseases & National Engineering Research Center of Oral Biomaterials and Digital Medical Devices, Peking University School and Hospital of Stomatology, Beijing, PR China; 4 Center for Digital Dentistry, Peking University School and Hospital of Stomatology & National Center for Stomatology & National Clinical Research Center for Oral Diseases & National Engineering Research Center of Oral Biomaterials and Digital Medical Devices, Beijing, PR China

**Keywords:** Dynamic navigation, Endodontic surgery, Accuracy, Root-end resection

## Abstract

**Background:**

The operation accuracy and efficiency of dynamic navigated endodontic surgery were evaluated through in vitro experiments. This study provides a reference for future clinical application of dynamic navigation systems in endodontic surgery.

**Materials and methods:**

3D-printed maxillary anterior teeth were used in the preparation of models for endodontic surgery. Endodontic surgery was performed with and without dynamic navigation by an operator who was proficient in dynamic navigation technology but had no experience in endodontic surgery. Optical scanning data were applied to evaluate the length and angle deviations of root-end resection. And the operation time was recorded. T tests were used to analyze the effect of dynamic navigation technology on the accuracy and duration of endodontic surgery.

**Results:**

With dynamic navigation, the root-end resection length deviation was 0.46 ± 0.06 mm, the angle deviation was 2.45 ± 0.96°, and the operation time was 187 ± 22.97 s. Without dynamic navigation, the root-end resection length deviation was 1.20 ± 0.92 mm, the angle deviation was 16.20 ± 9.59°, and the operation time was 247 ± 61.47 s. Less deviation was achieved and less operation time was spent with than without dynamic navigation (*P* < 0.01).

**Conclusion:**

The application of a dynamic navigation system in endodontic surgery can improve the accuracy and efficiency significantly for operators without surgical experience and reduce the operation time.

**Supplementary Information:**

The online version contains supplementary material available at 10.1186/s12903-024-04306-6.

## Background

Endodontic surgery is an important means of preserving teeth [1, 2]. Endodontic microsurgery is currently widely chosen by clinicians because it can effectively reduce postoperative pain/edema, accelerate wound healing and increase the success rate [3]. However, there are still challenges in the clinical application of endodontic microsurgery, including the accurate localization of the root end when the bone cortex is intact, the precise vertical resection of the root end, and the precise removal of the apical 3 mm [4–8].

Digital technology is widely applied in oral medicine. Static guides were introduced to assist in endodontic surgery to achieve accurate localization and resection of the root end [9]. However, static guides also have shortcomings, such as increasing treatment costs, affecting cooling water irrigation, and being difficult to use in patients with limited mouth opening [10–13]. Dynamic navigation can reduce the dependence on surgical experience and improve operation accuracy through real-time positioning and guidance of moving targets, and it is applicable for a wider range of patients than static guides. For example, patients with limited mouth opening or those who require posterior tooth apical surgery often face difficulties in achieving proper positioning of the surgical template [14–16]. At present, dynamic navigation has been widely used in implantology [17–19], and it has also been applied to locate calcified root canals in endodontics or remove fiber posts [20–22]. However, there have been few studies on the accuracy of using dynamic navigation in endodontic surgery, and most related studies have been clinical case reports [23–25]. To provide a reference for clinical application of dynamic navigation in endodontic surgery, it is necessary to evaluate the operation accuracy.

Thus, the influence of dynamic navigation on the operation accuracy and duration of endodontic surgery was evaluated using an in vitro plaster model and the results were compared with those of freehand surgery.

## Materials and methods

### Preparation of dentition models for endodontic surgery

**Fig. 1:**
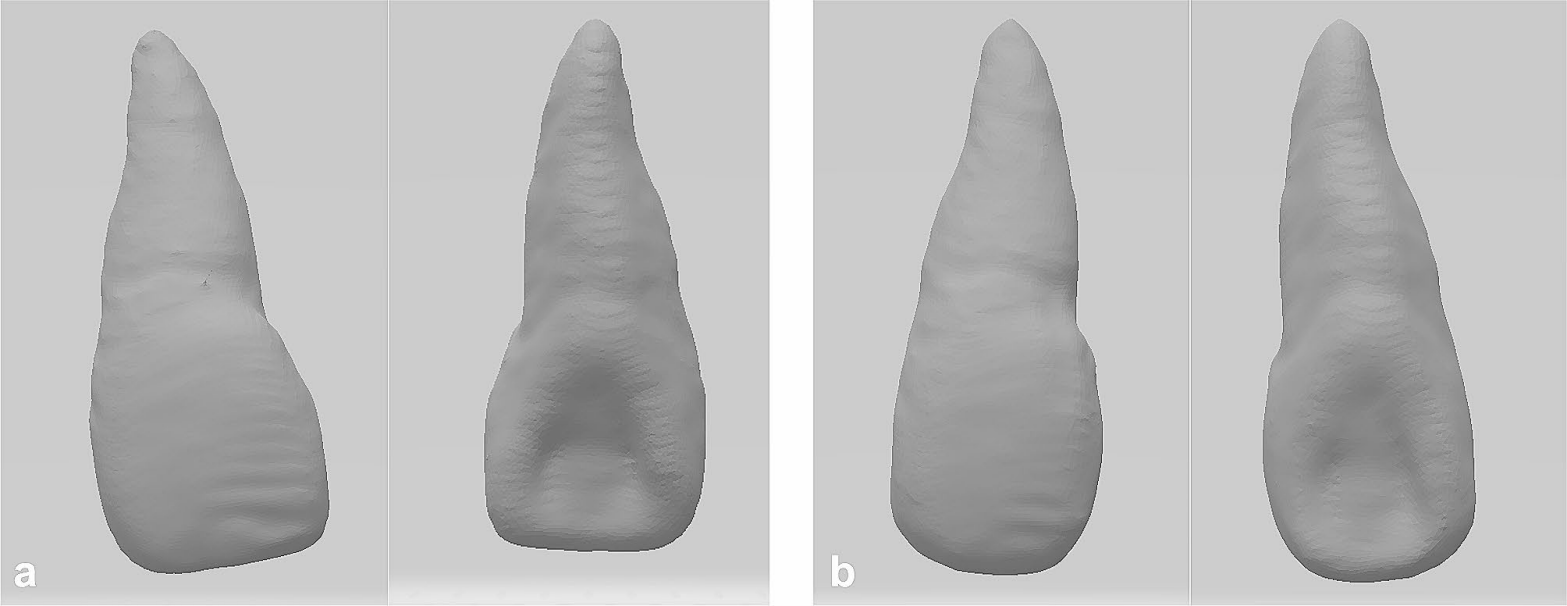
Data of the tooth before and after modification. (**a**) Optical scanning data of the extracted maxillary anterior tooth; (**b**) Modified tooth data for 3D printing

An extracted maxillary anterior tooth with less than 10° of root curvature, an intact root and a closed apex was optically scanned using a three-dimensional scanner (Activity 880, Smart Optics, Bochum, Germany). Rhino 7 software (McNeel, Seattle, WA, USA) was used to modify the tooth data so that the crown shape could fit the standardized dentition model (Fig. 1).

The modified tooth data were printed using an Objet30 Prime printer (Stratasys, Eden Prairie, MN, USA) and VeroClear resin (Stratasys, Eden Prairie, MN, USA). According to the sample size of the study on locating calcified root canals using dynamic navigation on 3D-printed teeth [26], 40 resin teeth with the same morphology were made.

The resin teeth were divided into the experimental group (with dynamic navigation) and the control group (freehand), with 20 teeth in each group. Sawdust and plaster (Heraeus Kulzer, Hanau, Germany) were used to simulate the alveolar bone and cast the dentition models.

We referred to studies similar to our research that applied dynamic navigation technology in the clinical treatment of endodontic diseases, one of which applied dynamic navigation technology for root canal localization with a sample size of 10 for dynamic navigation system group and freehand group [26]. In order to obtain more accurate results, we chose twice the sample size, which is 20 for.

### Design and operation of dynamic navigation-guided endodontic surgery

**Fig. 2:**
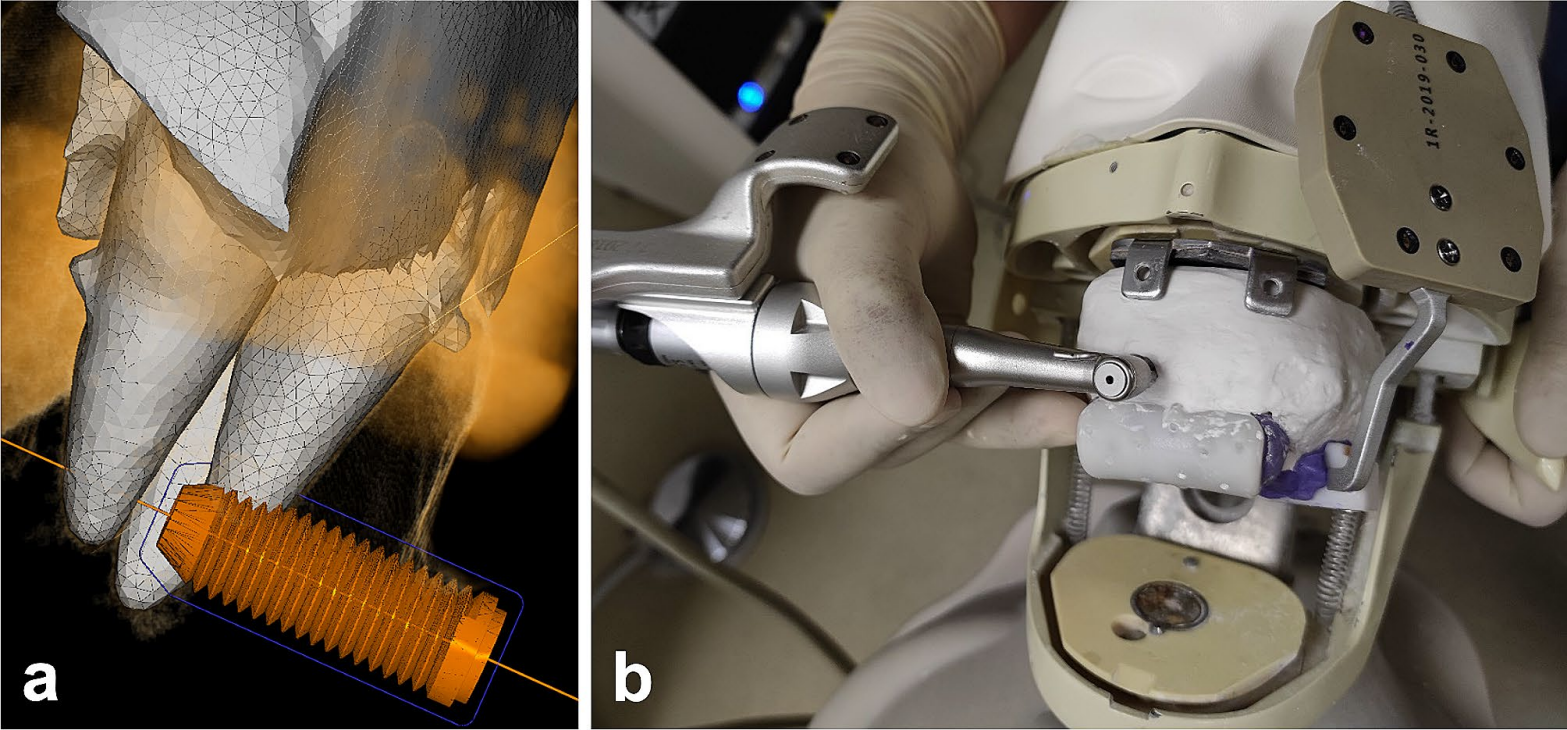
Approach design and operation with dynamic navigation. (**a**) Design of the endodontic surgery approach in dynamic navigation software; (**b**) Apical fenestration and resection under the guidance of dynamic navigation

For endodontic surgery under the dynamic navigation (DCARER, Suzhou, China), silicone rubber impression material (HUGE, Shanghai, China) was mixed evenly and placed into the registration device. The registration device was pressed on the dentition model. Cone-beam computed tomography (CBCT, New Tom VGi, QR Corporation, Verona, Italy) scanning was performed after the silicone rubber was fully cured. Then, the CBCT data were imported into the dynamic navigation software. An implant with a diameter of 4.5 mm and a non-tapered cylinder was used to design the approach of the trephine. The planned position of the trephine was set on the sagittal and axial planes according to the following criteria in order to vertically resect the apical 3 mm: (1) Depth: the trephine front exceeded the deepest boundary of the resin tooth. (2) Excision site: the distance between the central axis of the trephine and root end was 0.75 mm, where the upper edge of the trephine was 3 mm from the root end. (3) Angle: the angle between the central axis of the trephine and the long axis of the tooth was 90° (Fig. 2A). In this experiment, the long axis of the tooth was defined by the line between the anatomical apex and the geometric center of the axial surface of the tooth neck.

After calibrated, the handpiece was used to register the dentition model with the CBCT data. 0.6 mm was set as the length deviation tolerance, and 5° was set as the angle deviation tolerance. A resident physician with over 5 years of clinical experience in endodontic treatment, who has assisted in more than 20 apical surgery cases and completed one apical surgery under supervision completed 20 models of apical fenestration and resection under dynamic navigation (Fig. 2B). This operator is also skilled in dynamic navigation technology. During the operation, the length and angle deviations were displayed in real time. As the trephine was drilled deeper, the dynamic navigation system displayed the current remaining depth continuously. When it was prompted that the remaining depth reached 0.0 mm, the root-end resection was completed. The operation time was recorded for apical fenestration and resection.

### Design and operation of endodontic surgery without dynamic navigation

For the freehand operation, CBCT scans were directly performed on the maxillary dentition model. The operation path was designed by the same operator according to the CBCT data. A peripheral probe was used to locate the drilling site on the surface of the dentition model. 20 models of endodontic surgery was completed by the same resident physician without dynamic navigation using a high-speed fissure bur (1.6 mm in diameter). The operation time was recorded for apical fenestration and resection.

### Postoperative evaluation of root-end resection length and angle deviations

**Fig. 3:**
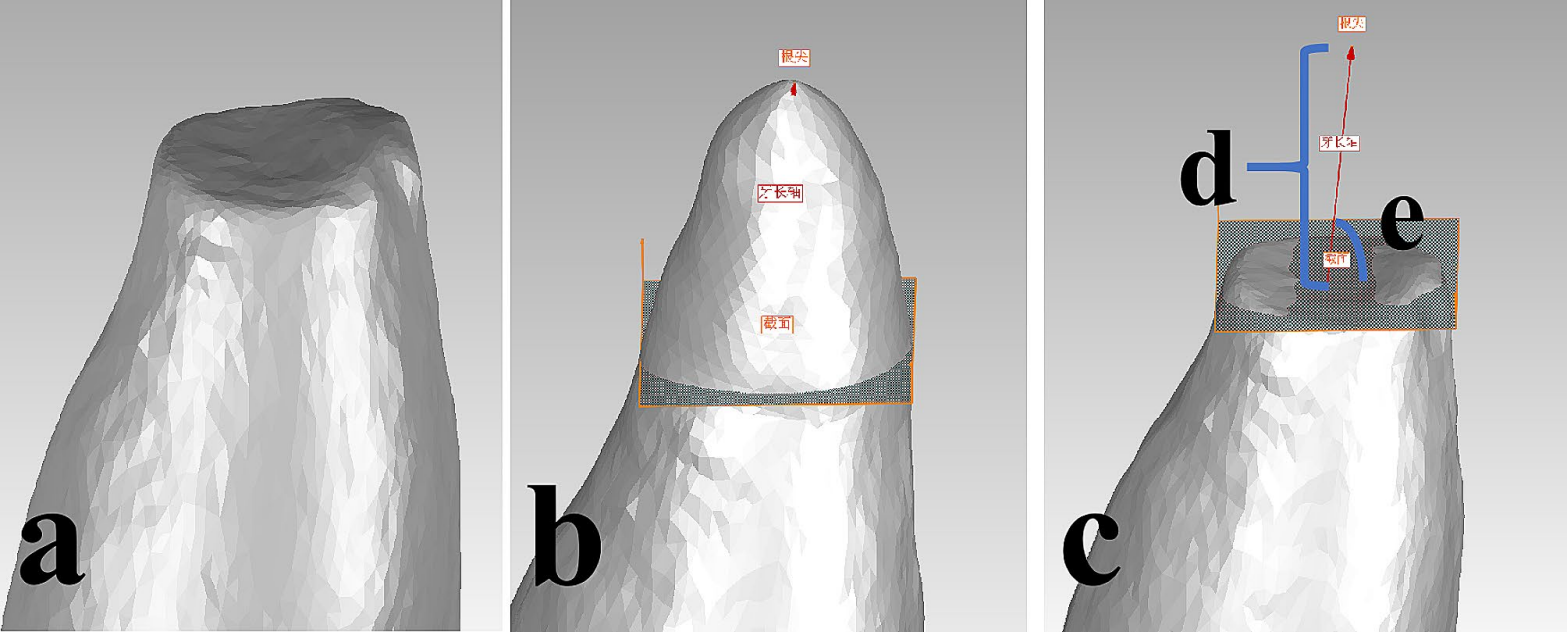
Evaluation of root-end resection length and angle deviations. (**A**) Postoperative optical scan data of resin teeth; (**B**) Determine The best-fitting registration based on the overlapping parts; (**C**) Comparison of the preoperative and postoperative optical scan data of resin teeth; (**D**) Length of the root-end resection; (**E**) Angle of the root-end resection

After the operation, the resin tooth was removed from the dentition model and optically scanned. In Geomagic Control software (Geomagic, Morrisville, NC, USA), an evaluator skilled in endodontic surgery and navigation (who was not the designer of the navigation approach or the operator) compared the preoperative and postoperative optical scan data of resin teeth from both groups in a blinded manner (Fig. 3A). The best-fitting registration was determined based on the overlapping parts (Fig. 3B). The postoperative root-end resection plane was obtained using best-fitting reconstruction on the root section of the postoperative tooth. The distance between the anatomical apex along the long axis of the tooth and the root-end resection plane was measured and compared with 3 mm to calculate the root-end resection length deviation (Fig. 3C), (Fig. 3D). The angle between the long axis of the tooth and the root-end resection plane was measured and compared with 90° to calculate the root-end resection angle deviation (Fig. 3E).

The evaluator measured the deviations of root-end resection length and angle for all samples by the same method again 2 weeks later. The samples were randomly sorted during the measurement. The intra-evaluator consistency test was carried out on the two measurement results, and the first evaluation values were taken for the final analyses.

### Statistical analysis

The data were imported into SPSS software (IBM, Chicago, IL, USA), and an intra-evaluator consistency test was carried out through the intraclass correlation coefficient (ICC). A normality test was carried out with the Shapiro-Wilk test, which showed that the data followed a normal distribution. T tests were applied to evaluate the length and angle deviations of root-end resection and operation time between the two groups.

## Results

The ICC was 0.965, showing good self-consistency. The differences in the length and angle deviations between the two groups were statistically significant (*P* < 0.01).

**Fig. 4:**
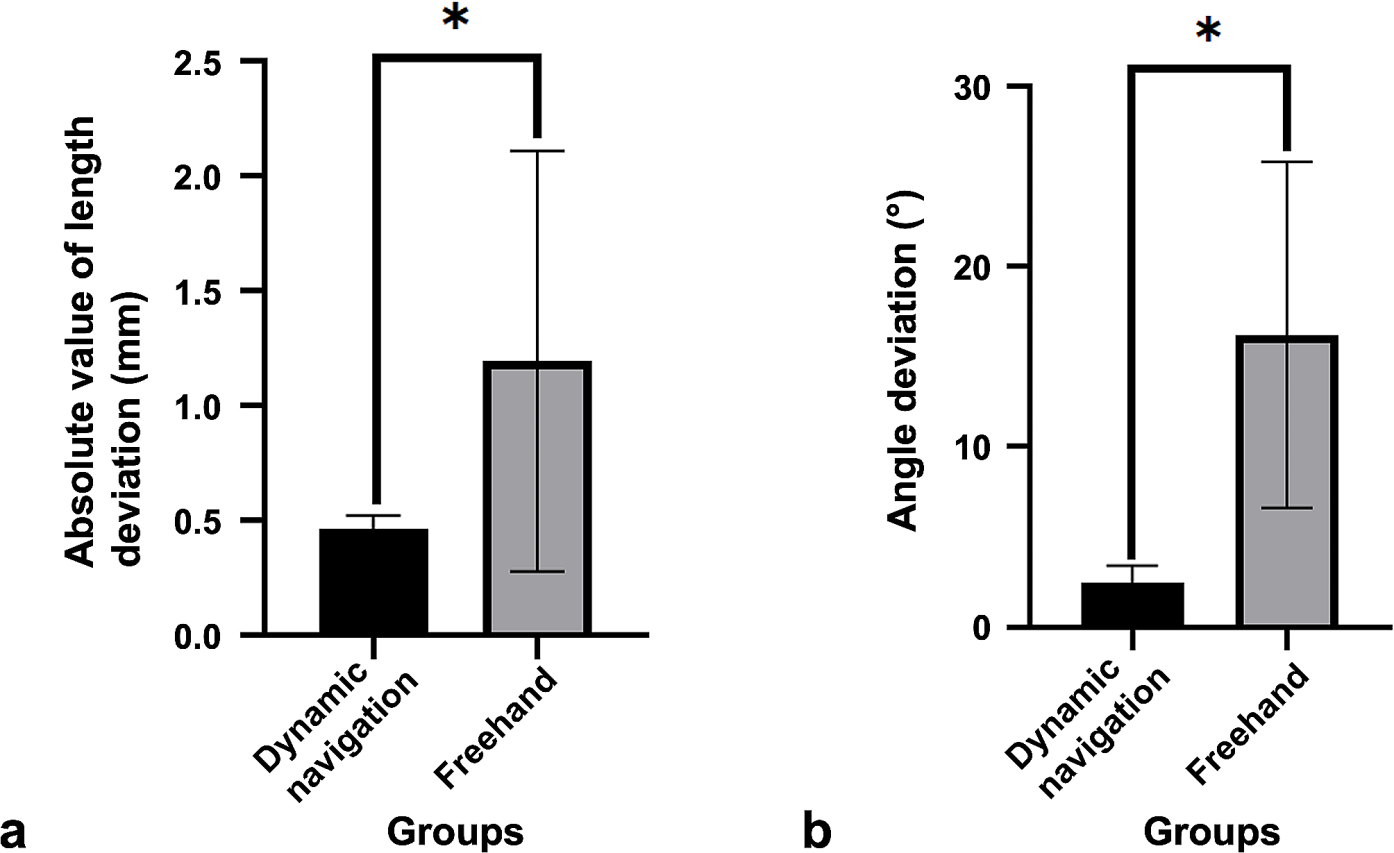
Deviation of root-end resection between the dynamic navigation group and the freehand group. (**A**) Absolute value of length deviation; (**B**) Angle deviation. *: The difference between the groups was significant (*P* < 0.01)

The deviations of the root-end resection are shown in Fig. 4. The absolute value of the length deviation was 0.46 ± 0.06 mm under the dynamic navigation. The absolute value of the length deviation was 1.20 ± 0.92 mm in the freehand group. The absolute value of the length deviation of root-end resection under the dynamic navigation was 0.74 mm (62%) lower than that in the freehand group (*P = 0.001* < 0.01) (Fig. 4A). The root-end resection angle deviation was 2.45 ± 0.96° under the guidance of dynamic navigation, while the angle deviation was 16.20 ± 9.59° without guidance. The angle deviation of root-end resection under dynamic navigation was 13.75° (85%) lower than that without guidance (*P = 1.725E-7* < 0.01) (Fig. 4B).

**Fig. 5:**
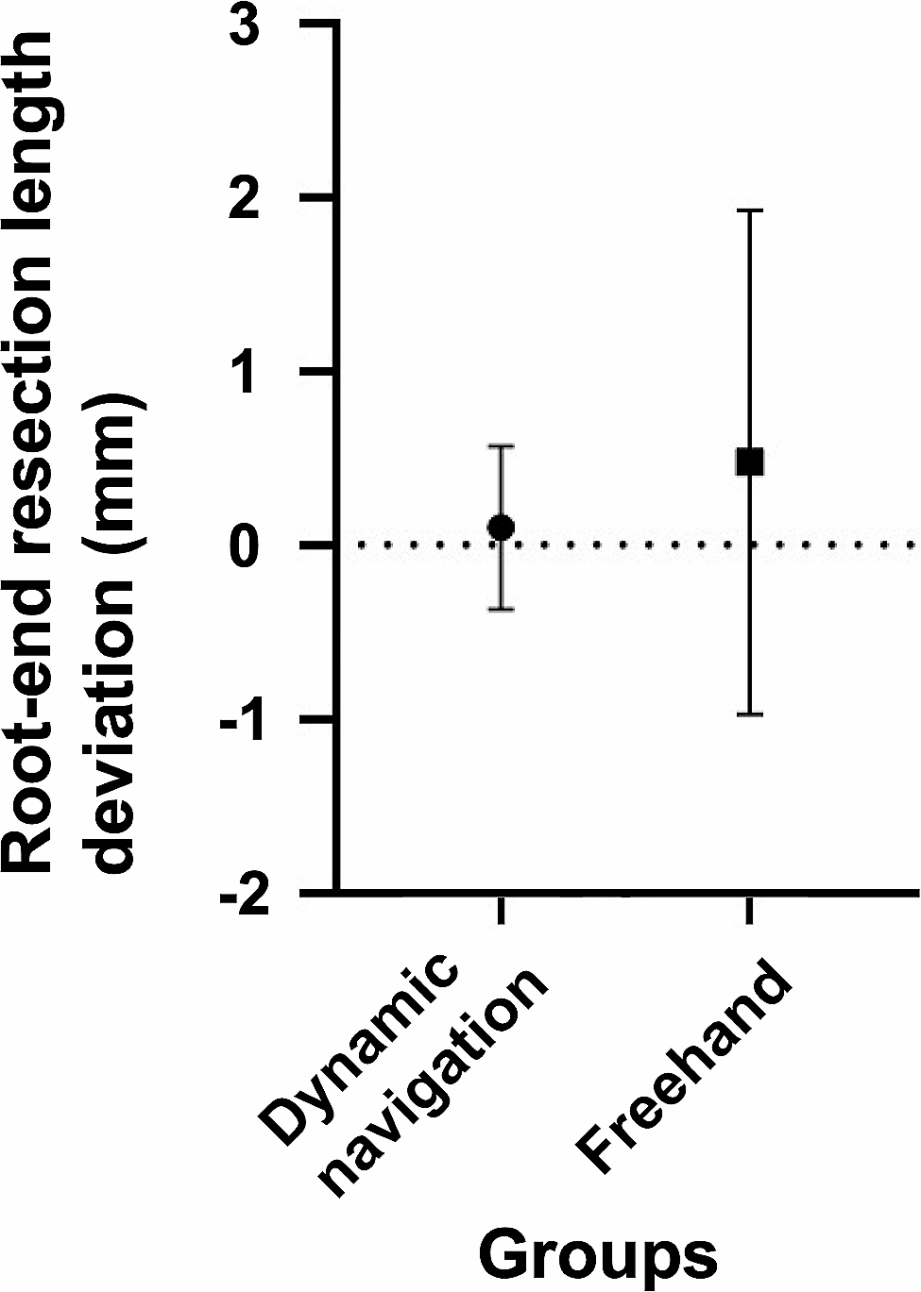
Distribution of root-end resection length deviation of the dynamic navigation group and the freehand group

The distribution of the root-end resection length deviation is shown in Fig. 5. The standard deviation in the dynamic navigation group was 0.47 mm, and it was 1.45 mm in the freehand group. The results showed that the variations in length and angle deviations were significantly reduced using dynamic navigation compared to freehand. The root-end resection process is more stable and controllable when guided by dynamic navigation than when performed freehand.

**Fig. 6:**
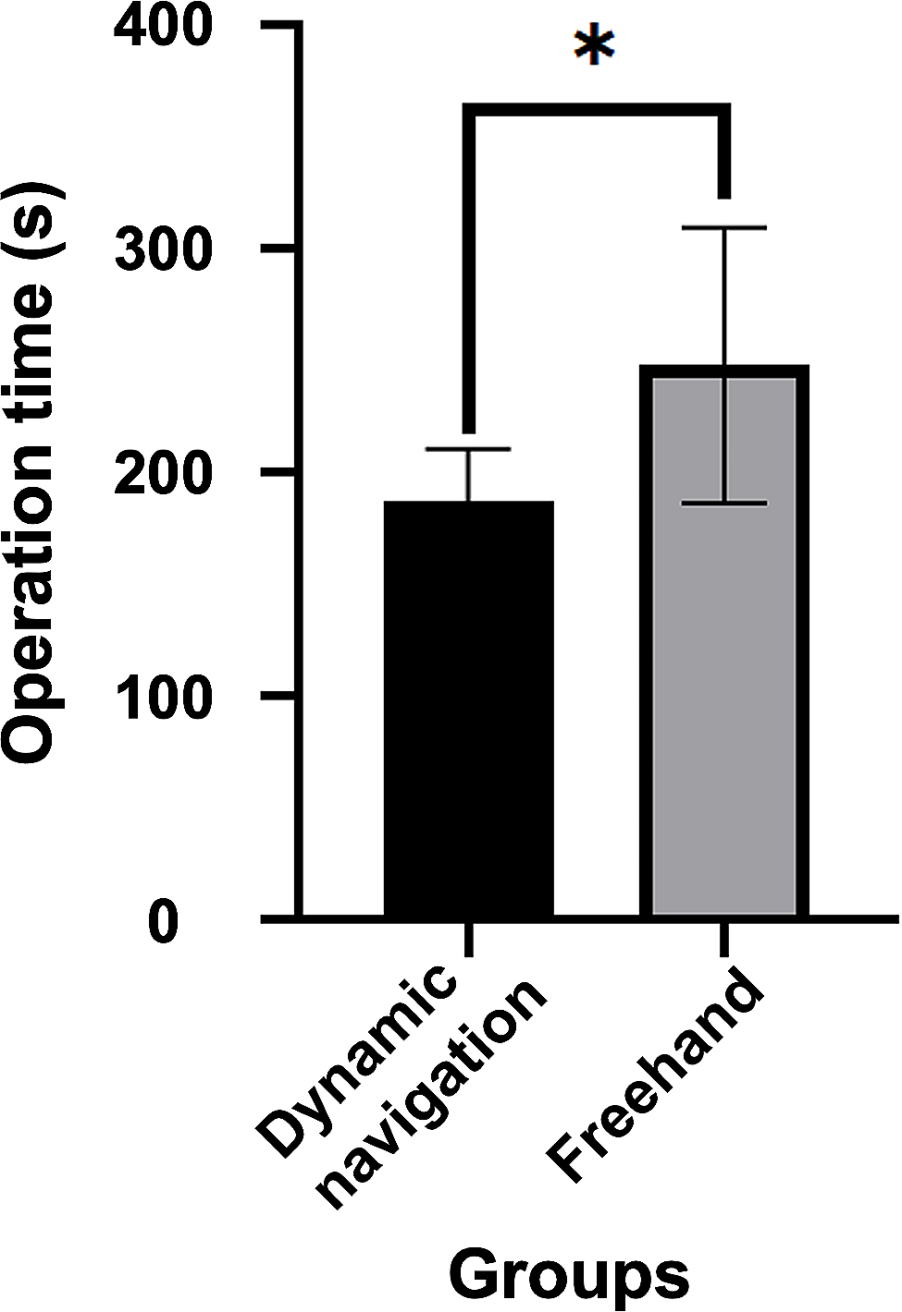
Operation time in the dynamic navigation group and the freehand group. *: The difference between the groups was significant (*P* < 0.01)

Under dynamic navigation, the average operation time from bone fenestration to root-end resection was 187 ± 22.97 s and that without guidance was 247 ± 61.47 s. There was a statistically significant difference in the operation time (*P* < 0.01) (Fig. 6).

## Discussion

Endodontic microsurgery has further expanded the indications and increased the success rate [27]. However, the success of endodontic surgery is highly dependent on the experience of the operator especially for difficult cases [28, 29].

In recent years, the application of dynamic navigation in stomatology has attracted increasing attention and has been used in implantology, as well as for the localization of calcified root canals and removal of fiber posts in endodontic treatment. However, there have been few studies on the accuracy of using dynamic navigation in endodontic surgery. In this study, dynamic navigation was successfully applied in endodontic surgery on a standardized dentition model and effectively improved the accuracy and efficiency of the operation.

### Significance of accurate root-end resection in endodontic surgery

Precise resection of the root end is one of the key steps in endodontic surgery. At present, there is no unified regulation on root-end resection length. Apical anatomical research has shown that at least 3 mm of the apex should be resected to ensure the removal of 98% of apical ramifications and 93% of lateral canals [3, 30]. Resecting 3 mm of the root end facilitates the removal of apical lesions and granulation tissues. The remaining root length after resecting 3 mm of the apex is 7–9 mm, which can provide sufficient strength and stability for the tooth.

As for the root-end resection angle, endodontic surgery requires that the approach should be as perpendicular as possible to the long axis of the tooth [3, 31, 32]. This ensures the smallest possible fenestration without creating a sharp section and can help to reduce the possibility of incomplete root-end resection. At the same time, this type of resection is convenient for the operator to determine the direction of the root canal and thus reduces the risk of root canal perforation during retrograde preparation. Also, vertical root-end resection reduces the number of exposed dentinal tubules [31]; thus, the possibility of retrograde bacterial infection through dentinal tubules is reduced. Additionally, this approach reduces the diameter of the section, which can reduce the possibility of microleakage around the periphery of retrograde filling materials [33]. Previous studies have indicated that the success rate of endodontic surgery is higher with a smaller root-end resection plane [32].

### Construction of an in vitro dentition model for endodontic surgery

In this study, a standardized 3D-printed resin tooth was used to construct the in vitro dentition model for endodontic surgery. Previous research indicated that root morphology may have an impact on the accuracy of endodontic surgery [34]. By unifying the anatomical morphology of the teeth, this study ensured that every operation was carried out on teeth with the same anatomical morphology. In addition, it ensured a unified standard in the measurement and analysis of root-end resection length and angle, which can be analyzed according to the same tooth long axis and the same anatomical apex.

### Role of the trephine in endodontic surgery

In this study, a trephine that was 4.5 mm in diameter was used for apex localization and resection. The application of a trephine can allow apex localization and root-end resection to be achieved in a single operation, which is an efficient and convenient tool in endodontic surgery [35]. The positioning accuracy of the trephine is not lower than that of the traditional fissure bur [29, 36]. Trephines were first used in implantology for the extraction of failed implants and for autologous bone grafting procedures [37–39]. However, when a trephine is used in endodontic surgery, if the apex is not localized accurately or if the drilling angle is not perpendicular to the long axis of the tooth, operative corrections cannot be made during the procedure. Thus, a trephine should be used under guidance in endodontic surgery. Therefore, in this study, the trephine was only used under dynamic navigation, and a fissure bur was used in the freehand group.

The diameter of trephines used in previous studies on endodontic surgery ranged from 4.21 mm to 4.46 mm [10, 34, 40]. In this study, a trephine with a diameter of 4.5 mm was used to ensure a clear surgical field during endodontic surgery under the premise of controlling bone damage. The size of the bone fenestration was sufficient to accommodate retrograde preparation, retrograde filling and use of the microscopic oral mirror [10]. Other studies have indicated that a trephine with a diameter of 4.4 mm shows good adaptability for use in treating apical lesions of different sizes and roots of different tooth positions and diameters [40].

### Influence of dynamic navigation technology on the precision of endodontic surgery in vitro

The results of this study showed that with the guidance of dynamic navigation, operators with no experience in endodontic surgery can accurately complete fenestration, apex localization and root-end resection in endodontic surgery. The dentition model in this research simulates a case where there is no sinus opening on the labial or buccal surface and the apex is deep, which increases the difficulty of completing the endodontic surgery without guidance. In this study, without guidance, the absolute value of the length deviation reached 1.20 ± 0.92 mm and that of the angle deviation reached 16.20 ± 9.59°.

Excessive length and angle deviations during endodontic surgery increase the risk of damage to adjacent structures. A previous study indicated that in 8.35% of cases, the apex was less than 1.00 mm from the mandibular canal, and in 1.79% of cases, the root end contacted or invaded the mandibular canal [7]. The application of dynamic navigation significantly reduces positioning deviation. In this study, under the guidance of dynamic navigation, the absolute value of length deviation was 0.46 ± 0.06 mm, and the angle deviation was 2.45 ± 0.96°, demonstrating greater accuracy than in the freehand group.

The results of this study show satisfactory positioning accuracy with the introduction of dynamic navigation. The results of previous studies using a trephine with a static guide for endodontic surgery indicated that the mean length deviation was 0.92 ± 0.60 mm and that the average root-end resection was 2.7 mm [10]. The length deviation in this study was 0.46 ± 0.06 mm, indicating higher accuracy.

In this study, the apical surgery was performed by a doctor who had less surgical experience but was proficient in dynamic navigation technology. This highlights the potential of dynamic navigation technology to improve the precision of apical surgery for young or less experienced doctors. However, it is important to note that if the same procedure were performed by a doctor with extensive experience in apical surgery, he or she might complete the procedure more quickly with or without relying on dynamic navigation technology.

Another limitation of this study is that it utilized the data provided by the evaluator for the first measurement, instead of calculating the average of the preoperative and postoperative data. The purpose of this approach was to better align with the clinical reality. Although there were no statistically significant differences between the preoperative and postoperative data, using the average of the two measurements would have been more effective in reducing errors associated with manual measurements and providing a more accurate reflection of the true data. In future research on dynamic navigation technology, we will be inclined to use the average of the two data sets.

This study focused on evaluating whether dynamic navigation technology can improve the accuracy of apical surgery for a single operator. It is a prospective study aimed at paving the way and laying the foundation for the future application of dynamic navigation technology in the field of microsurgical apical surgery. It aims to provide direction for future research in which we will conduct broader and more extensive studies on dynamic navigation technology, specifically focusing on whether this technology can enable less experienced clinicians to achieve high surgical accuracy. As expressed by the operator who used dynamic navigation technology to perform a case of ex vivo microsurgical apical surgery, it is a digital technology that allows more junior clinicians to explore complex surgical pathways.

## Conclusions

Considering the problems of difficult positioning and insufficient accuracy in endodontic surgery, this study constructed a dentition model with standardized 3D-printed resin teeth, used the dynamic navigation technology to guide the trephine, and preliminarily studied the effect of dynamic navigation technology on endodontic surgical outcomes. The results showed that dynamic navigation can significantly improve the operation accuracy of clinicians without operative experience, reduce the root-end resection length and angle deviations, and reduce the operation time. The results of this study provided a reference for the application of dynamic navigation in endodontic surgery. In further studies, systematic clinical research should be carried out on the application of dynamic navigation in endodontic surgery.

### Electronic supplementary material

Below is the link to the electronic supplementary material.

Supplementary Material 1

## Data Availability

Data is provided within the Supplementary material (data.xlsx).

## Abbreviations

CBCT: Cone-Beam Computed Tomography
ICC: Intraclass Correlation Coefficient (ICC)
3D: Three Dimensional
